# HIF-1α as a Target Molecule in the Use of Triazino-Indole Derivative on the Acoustic Trauma Model

**DOI:** 10.3390/audiolres11030034

**Published:** 2021-07-15

**Authors:** Vladimir L. Pastushenkov, Leonid G. Buynov, Maksim S. Kuznetsov, Vladimir V. Dvorianchikov, Lev A. Glaznikov, Aleksandr L. Pastushenkov

**Affiliations:** 1 LLC “Farm-Trisan”, 191015 St. Petersburg, Russia; pastprof@mail.ru; 2 Department of Medical and Valeological Disciplines, The Herzen State Pedagogical University of Russia, 191015 St. Petersburg, Russia; 3 Department of Otorhinolaryngology, Military Medical Academy, 191015 St. Petersburg, Russia; mskuznecov2@mail.ru (M.S.K.); vmedalor@mail.ru (V.V.D.); glaznikov@mail.ru (L.A.G.); 4 Department of Pharmacy and Pharmacology, North-Western State Medical University Named after I.I. Mechnikov, 191015 St. Petersburg, Russia; palunov@mail.ru

**Keywords:** hypoxia, hypoxia-inducible factor, HIF-1α, acoustic trauma, antihypoxants, triazino-indole, expression

## Abstract

The effect of triazino-indole derivative (Trisan) on hypoxia-inducible factor (HIF) expression level in the organ of Corti, when administering it for therapeutic and preventive purposes, was investigated using an acoustic trauma model in experimental animals (female F1 hybrids of CBA and C57BL/6 lines). Cytoflavin was used as a comparator product. Study product Trisan (1% solution) was injected intravenously, intramuscularly and intraperitoneally, in the dose of 5, 7 and 10 mg/kg 2 h after the acoustic trauma for therapeutic purposes and in the dose of 5, 7 and 10 mg/kg for 3 days before the acoustic trauma for preventive purposes. IHC methods were used to investigate the organ of Corti. Trisan was observed to increase HIF expression in hair cells and neurons of the spiral ganglion in case of acoustic trauma. Depending on the dose, the increased HIF-1 expression in hair cells and spiral ganglion occurred both after therapeutic and preventive use of Trisan. Maximum HIF expression in hair cells and ganglion was noted at the therapeutic and preventive drug dose of 10 mg/kg. Following experimental results, we conclude that the otoprotective effect of triazino-indole derivative is realized via its effect on HIF metabolism, which makes it a target molecule for the drug.

## 1. Introduction

In the modern world, the prevalence of noise-induced hearing loss has arguably reached epidemic proportions [1]. Prior research has demonstrated that exposure to high levels of noise results in microcirculation disturbance in the internal ear and, subsequently, in hypoxia in the organ of Corti [2]. This results in accumulation of active oxygen and nitrogen forms, so-called oxidative stress and programmed and/or necrotic cell death [3]. In addition to damage to hair cells, there is also irreversible damage to neurons in the auditory path [4]. This pathology is defined in the literature as “cochlear synaptopathy” and was reflected in several experimental animal studies [5].

According to prior research, hypoxia resistance of hair cells in the organ of Corti is realized through the path associated with an HIF molecule [6]. It was first identified and investigated by Gregg Semenza and researchers from the Johns Hopkins University in Baltimore in 1992 [7]. They were awarded a Nobel Prize for physiology and medicine for their studies in 2019.

HIF belongs to a basic helix–loop–helix (bHLH) class of transcription proteins found in all mammalian cells and tissues. It is required for transcription activation in cells under hypoxia conditions through the erythropoietin gene [8]. The HIF molecule exists mainly in heterodimer form [9]. Its composition includes an oxygen-independent subunit HIF-1β and three oxygen-dependent transcription-active subunits, of which HIF-1α and HIF-2α are responsible for improvement of cell adaptation to hypoxia. HIF-3α is a negative regulator of hypoxia-inducible genes. It has been reported that HIF-3α isoform can inhibit the action of HIF-1/2α [10]. The HIF-1α quantity in the cell is maintained at the low level in normoxia but increases greatly in hypoxia [11]. HIF is an oxygen homeostasis regulator with hundreds of hypoxia-inducible target genes, protein products of which play a significant role in angiogenesis [12], energy metabolism [13], regulation of apoptosis and necrosis paths [14], erythropoiesis [15] and iron homeostasis [16].

The HIF-1α quantity is regulated by VHL protein (Von Hippel–Lindau), which is involved in its ubiquitination and proteasome degradation due to E3 ligase [17]. Hydroxylation of HIF-1α molecule with enzyme prolyl hydroxylase (PHD) is required for interaction with VHL. The reaction involves molecular oxygen, iron ions and ascorbic acid. If the quantity of one of these components is insufficient, hydroxylation reaction becomes impossible which results in the increase of HIF-1α quantity [18,19].

Our research involving an acoustic trauma model demonstrates that HIF-1α expression level can indeed be used to assess the degree of tissue hypoxia [20].

Currently, there are ongoing efforts to identify tools for pharmacologic correction of hypoxic damage of the organ of Corti caused by acoustic trauma using HIF as their target what formed the base of the present study.

*Objective of the study.* To assess hypoxia-inducible factor HIF-1α expression in cells of the organ of Corti and its morphologic changes using an acoustic trauma model while administering triazino-indole derivative.

## 2. Materials and Methods

The experiments were performed using 123 female mice of F1 hybrids of CBA and C57BL/6 line with weight of not less than 17 g aged 4–12 weeks. The mice were delivered from the laboratory animal farm of the Russian Academy of Medical Sciences “Rappolovo” (Leningrad region, Russia). The experiments were performed 14 days after the animals adjusted to the vivarium conditions. All experimental research was performed in accordance with the requirements of the “European Convention for the Protection of Vertebrate Animals used for Experimental and other Scientific Purposes” (Strasbourg, 1996), rules of laboratory practice (Order of the Ministry of Social Development of the Russian Federation dated 23 August 2010) and methodical recommendations on performing preclinical studies. Euthanasia of mice was carried out using a lethal dose of anaesthesia used in the experiment, i.e., intraperitoneal injections of Telazol (50–70 mg/kg).

We studied a new triazino-indole derivative (Trisan) 3-[(2-morpholinoethylthio]-5H-1,2,4- triazino [5,6-b] indole dihydrochloride, monohydrate. It was first investigated by Doctor of Medical Sciences, Professor L.V. Pastushenkov at the Department of Pharmacology at the Kirov Military Medical Academy. Cytoflavin—having antioxidant, antihypoxic actions and allowed in ENT-practice for treatment of acute and chronic sensorineural hearing loss—was used as a comparator product [21].

When modelling acute acoustic trauma, we exposed an animal to white noise at the level of 107 dB (sound pressure level) and frequency band of 3–100,000 Hz (GRC Concord 1390-B Noise Generator, USA) for 3 h. The noise level of 107 dB corresponded to the level measured using the Brüel & Kjaer measuring system (Denmark): 6.5 mm of the calibration microphone of type 4145, preamplifier 2633 and measuring amplifies 2606 in the location point of the tympanic membrane of the test ear.

Animals were injected with the study product Trisan, 1% solution, intravenously, intramuscularly and intraperitoneally with single-use syringes. The ready-made 1% solution of Trisan was diluted with physiological saline (1 mL of 1% solution of Trisan and 9 mL of 0.9% physiological saline) for convenient injection. The drug was administered in the dose of 5, 7 and 10 mg/kg 2 h after the acoustic trauma for therapeutic purposes. The drug was administered in the dose of 5, 7 and 10 mg/kg for 3 days before the acoustic trauma for preventive purposes. The comparator product Cytoflavin was diluted and injected in the dose of 1.7 mL/kg by the same method. The experimental animals in the control group received physiological saline.

After completing the experiment, the organ of Corti was removed as part of the excised temporal bone, fixed with 10% normal (pH 7.4) formaldehyde solution for 12 h. The obtained fixed samples were decalcified by incubation in the rapid decalcification solution (Bio-Vitrum, St. Petersburg, Russia) for 30 min. Mice brain samples were processed using conventional techniques in the Excelsior AS tissue processor (Thermo Shandon Limited, Runcorn, UK), with isopropyl alcohol and paraffin wax. After that, a rotary microtome was used to prepare 5 μm thick cross-sections. Those cross-sections were then put on slides and coloured with haematoxylin and eosin.

The IHC tests were done with Autostainer 480S (Thermo Shandon Limited, Runcorn, UK), UltraVision Mouse HRP (Thermo Scientific, Waltham, MA, USA) and mouse monoclonal antibodies for HIF1 (Affinity Biosciences, 1:100 dilution, 90 min incubation time, with high-temperature sample pre-treatment at pH 6.0) according to the recommendations of equipment and reagent manufacturers. The IHC samples were scanned in Pannoramic Midi scanner (3DHISTECH Kft., Budapest, Hungary); the QuantCenter image analysis platform (3DHISTECH Kft., Budapest, Hungary) was used to calculate the HIF1-positive area (in %) of the modiolus and other areas of the brain. The colour was assessed using a mean cytochemical coefficient developed by the authors. The colour intensity in case of HIF-1α expression was designated with figures from 1 to 6.

## 3. Results

Table 1 presents the data on HIF expression level depending on the therapeutic and preventive dose assessed with the mean cytochemical coefficient.

**Figure 1:**
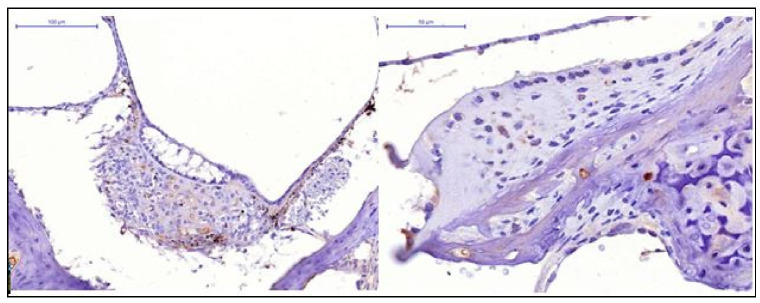
Absence of HIF-1 expression in the cells of the receptor apparatus, low HIF-1 expression in the cells of the spiral ganglion (**left**): control group, (**right**): Cytoflavin.

When analysing the experimental results of the specimens of the control and Cytoflavin groups (Figure 1), we noted absence of HIF-1 expression in the cells of the receptor apparatus, low HIF expression in cells of the spiral ganglion.

**Figure 2:**
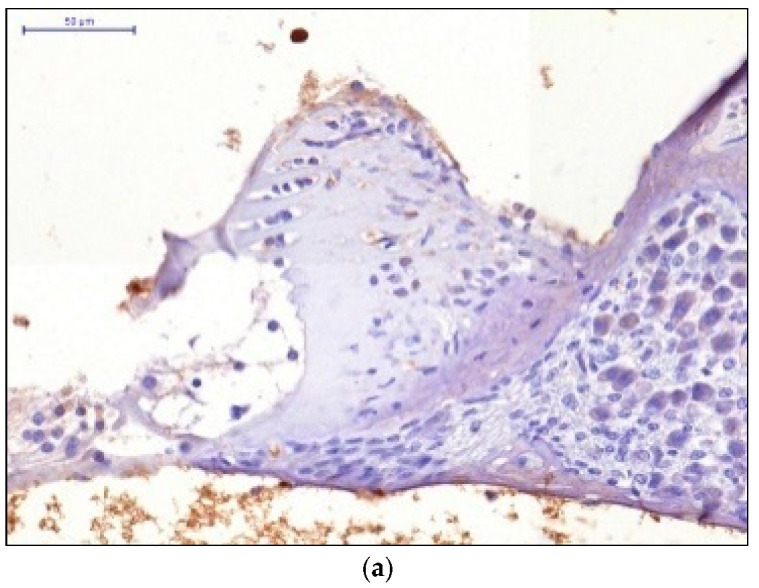

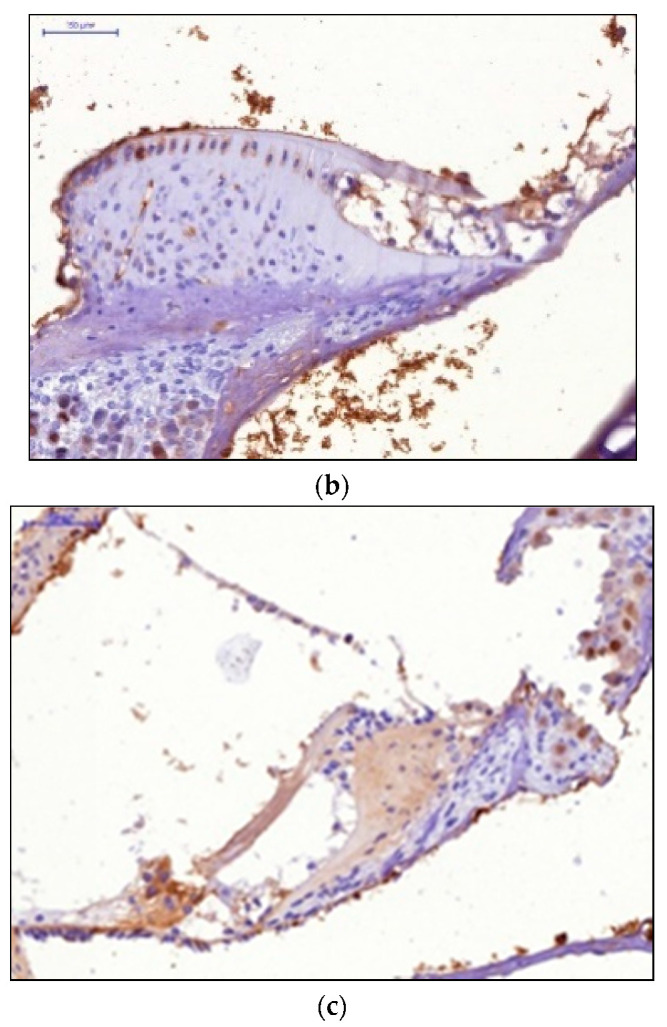
HIF-1 expression in the cells of the receptor apparatus and the cells of the spiral ganglion: (**a**) Therapeutic dose 5 mg/kg; (**b**) Therapeutic dose 7 mg/kg; (**c**) Therapeutic dose 10 mg/kg.

When the therapeutic dose of 5 mg/kg was administered, we observed low HIF-1 expression in the cells of the receptor apparatus and moderate HIF-1 expression in the cells of the spiral ganglion. When the therapeutic dose of 7 mg/kg was administered, we observed moderate HIF-1 expression in the cells of the receptor apparatus and the cells of the spiral ganglion. The pronounced HIF-1 expression was observed after administering the therapeutic dose of 10 mg/kg injected 2 h after the acoustic trauma (Figure 2).

**Figure 3:**
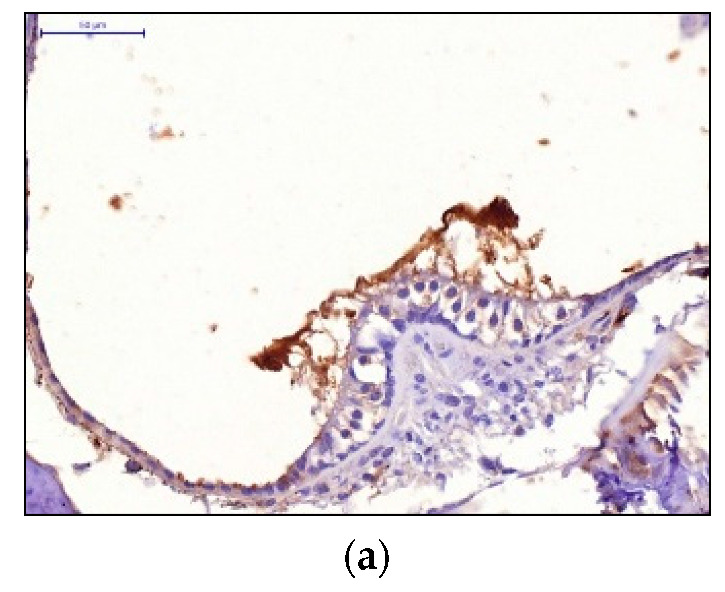

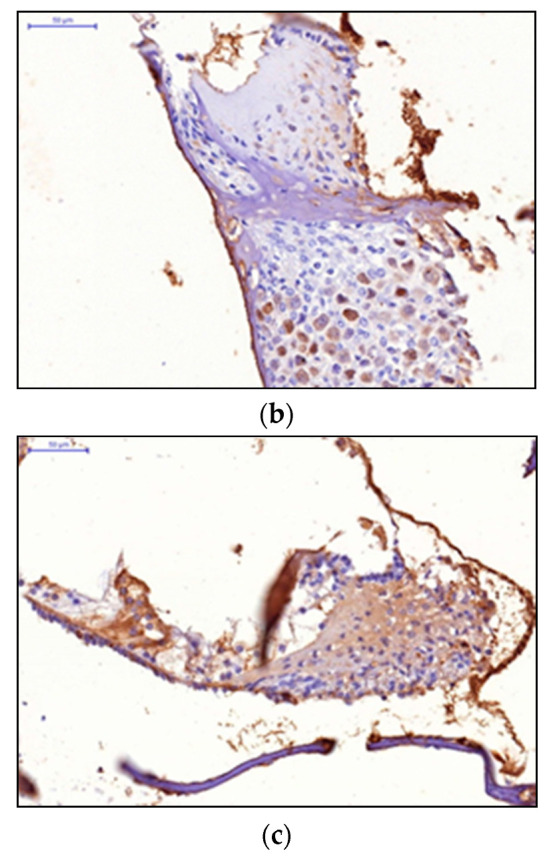
HIF-1 expression in the cells of the receptor apparatus and the cells of the spiral ganglion: (**a**) Preventive dose 5 mg/kg; (**b**) Preventive dose 7 mg/kg; (**c**) Preventive dose 10 mg/kg.

The preventive use of the drug 3 days before the acoustic load in the dose of 5 mg/kg resulted in moderate HIF-1 expression by the hair cells, low-level expression in the cells of the spiral ganglion. Administration of the dose of 7 mg/kg resulted in moderate HIF-1 expression in the cells of the receptor apparatus and the cells of the spiral ganglion. Injection of the dose 10 mg/kg resulted in pronounced HIF-1 expression in the cells of the receptor apparatus and the cells of the spiral ganglion (Figure 3).

In our study, we found that increased HIF-1 expression in the hair cells and spiral ganglion was observed depending on the increase of the drug dose both in case of the therapeutic and preventive use of triazino-indole derivative (Trisan). This coincided with electrophysiologic changes in experimental animals. This pattern suggests that HIF is indeed the target molecule for the drug and that the drug can regulate its level in the cells and neural pathways of the internal ear. The maximum HIF expression in the hair cells and ganglion was observed after administration of the preventive and therapeutic dose of 10 mg/kg.

## 4. Discussion

Prior literature contains reports of experiments demonstrating the use of drugs to increase HIF-1 expression. One of the methods for this is to block enzymes involved in HIF deactivation [21,22]. For instance, K.Y. HoWangYin et al. used human mesenchymal stem cells and observed inhibition of PHD2 enzyme which is involved in hydroxylation of HIF molecule what results in its proteolytic degradation. The authors hope than PHD2 inhibition and target gene induction of vascular endothelial growth factor through HIF-1α molecule can enhance therapy efficiency when treating patients with ischemic changes in the lower extremities [23,24]. Other authors used low-molecular-weight organic compounds of vegetable origin to activate HIF [25]. It has been demonstrated that PHD use iron as cofactors. This forms the basis for the mechanism of action of deferoxamine, which is iron chelator and prevents HIF degradation [26]. Dimethyloxalylglycine, which induces stabilization of HIF molecule, has the same properties [27,28]. The role of cobalt dichloride in increased HIF accumulation and transcription activity due to exclusion of iron and ascorbic acid from its metabolism has been demonstrated [29].

Recent studies have shown that microRNA, which regulate gene expression endogenously through the RNA-interference path, allow for selective action of HIF molecule, thereby increasing its stability [30]. A method for increasing HIF expression through modern genetic engineering tools using VP16-HIF-1α viruses is described [31].

According to the results of our study, triazino-indole derivative (Trisan) has an otoprotective action due to its effect on HIF expression level, most likely, because it blocks the enzymes involved in its degradation (prolyl hydrolases). This makes it possible to consider this drug as a promising pathogenetic agent for prevention and treatment of noise-induced hearing loss.

## 5. Conclusions

Basing on the results of our study and analysis of prior research, we conclude that triazino-indole derivative (Trisan) has an otoprotective action due to its effect on HIF expression level as a target molecule for the drug. As the dose of Trisan increases, there is an appropriate increase of HIF-1 expression in the hair cells and the spiral ganglion. We suppose that the mechanism of action of the drug is associated with blocking the enzymes involved in its proteosome degradation (prolyl hydroxylase).

## Figures and Tables

**Table 1:** The hypoxia-inducible factor (HIF) content in the organ of Corti structures when using different therapeutic and preventive regimens with the drug Trisan.

Treatment Type	Mean Cytochemical Coefficient	HIF Expression Based on the Findings of Immunohistochemical Investigation
Control	1	Absence of HIF-1 expression in the cells of the receptor apparatus, low-level HIF-1 expression in the cells of the spiral ganglion
Cytoflavin	1	Absence of HIF-1 expression in the cells of the receptor apparatus, low-level HIF-1 expression in the cells of the spiral ganglion
Trisan, treatment, 5 mg	2	Low-level HIF-1 expression in the cells of the receptor apparatus, moderate HIF-1 expression in the cells of the spiral ganglion
Trisan, treatment, 7 mg	4	Moderate HIF-1 expression in the cells of the receptor apparatus and the cells of the spiral ganglion
Trisan, treatment, 10 mg	5	Pronounced HIF-1 expression in the cells of the receptor apparatus, moderate HIF-1 expression in the cells of the spiral ganglion
Trisan, prevention, 5 mg	2	Moderate HIF-1 expression by the hair cells, low-level expression by the cells of the spiral ganglion
Trisan, prevention, 7 mg	4	Moderate HIF-1 expression in the cells of the receptor apparatus and the cells of the spiral ganglion
Trisan, prevention, 10 mg	6	Pronounced HIF-1 expression in the cells of the receptor apparatus and the cells of the spiral ganglion

## Data Availability

Not applicable.
